# Unexplained Variance in Hydration Study

**DOI:** 10.3390/nu11081828

**Published:** 2019-08-07

**Authors:** Colleen X. Muñoz, Michael Wininger

**Affiliations:** 1 Department of Health Sciences and Nursing, University of Hartford, West Hartford, CT 06117, USA; 2 Department of Rehabilitation Sciences, University of Hartford, West Hartford, CT 06117, USA; 3 Department of Biostatistics, Yale University, New Haven, CT 06510, USA; 4 Cooperative Studies Program, Department of Veterans Affairs, West Haven, CT 06516, USA

**Keywords:** hydration, water intake, obesity, modeling, database, NHANES, chronic disease, big data

## Abstract

With the collection of water-intake data, the National Health and Nutrition Examination Survey (NHANES) is becoming an increasingly popular resource for large-scale inquiry into human hydration. However, are we leveraging this resource properly? We sought to identify the opportunities and limitations inherent in hydration-related inquiry within a commonly studied database of hydration and nutrition. We also sought to critically review models published from this dataset. We reproduced two models published from the NHANES dataset, assessing the goodness of fit through conventional means (proportion of variance, R^2^). We also assessed model sensitivity to parameter configuration. Models published from the NHANES dataset typically yielded a very low goodness of fit R^2^ < 0.15. A reconfiguration of variables did not substantially improve model fit, and the goodness of fit of models published from the NHANES dataset may be low. Database-driven inquiry into human hydration requires the complete reporting of model diagnostics in order to fully contextualize findings. There are several emergent opportunities to potentially increase the proportion of explained variance in the NHANES dataset, including novel biomarkers, capturing situational variables (meteorology, for example), and consensus practices for adjustment of co-variates.

## 1. Introduction

Water intake and hydration status are evolving as increasingly important points of focus in far-reaching corners of medicine and public health. Investigators in search of phenotypic risk factors and as a possible strategy for mitigating disease burden and progression have targeted total water intake practices in myriad diseases including hyperglycemia [1,2], obesity [3,4,5,6,7,8], diabetes mellitus [5,6,9,10,11,12], metabolic syndrome [6,13], cardiovascular diseases [14,15,16,17,18], chronic kidney disease [5,19,20,21,22,23], cystic renal disease [24,25,26], and bladder cancer [27,28,29]. Given the extensive foundational research linking total water intake to other high-relevance morbidities, it is exciting that the National Center for Health Statistics collects water intake data. However, are we leveraging this resource optimally?

In this article, we explore model design in database-supported hydration inquiry. We contrast model diagnostics and explore optimization scenarios by reproducing two recently published regression models related to hydration. In particular, we look into regression models in hydration, published by others, by way of the model goodness of fit parameter, the R^2^. The coefficient of determination (0 ≤ R^2^ ≤ 1) is a standard measure for how well scatter data fit to their model regressor; it is the proportion of explained variation relative to total variation. A low R^2^ value indicates a large proportion of unexplained variance; a high R^2^ indicates that much of the variance observed in the data are explained by effects described in the model.

Our interest was to replicate two recently-published models in order to ascertain their goodness of fit. We also wanted to extend these published works by assessing the sensitivity of the model goodness to parameter selection. The models studied here are mutually similar but distinct, making use of different variables in both prediction and response, but both had a similar design in that regression models were designed to test specific hypotheses within a large, publicly available dataset. Our objective was to critically review the state of the art—and identify opportunities to enhance—database-driven inquiry into human hydration.

## 2. Methods and Results

### 2.1. Study Selection

We took as exemplars two recently published papers: Rosinger et al. [3] and Chang et al. [4]. Both groups leveraged the same dataset (National Health and Nutrition Examination Survey (NHANES) 2009–2012), tested for associations between hydration and body composition, were posed as population studies (i.e., weighted analyses), and utilized a parallel design, i.e., tandem linear and logistic regression. Naturally, these papers differ in terms of co-variate selection, dataset filtering, the selection of predictor versus response variable, and the age-adjustment of hydration status. Full methodologies are described in the original manuscripts, but in short summary: The Rosinger et al. study (*n* = 9528) utilized urine osmolality (URXOAV) as the response variable, with the following predictor variables: Age (RIDAGEYR) stratified 20–39, 40–59, and ≥60 years; gender (RIAGENDR); race–ethnicity (RIDRETH1) re-coded into three groups (Non-Hispanic White, Non-Hispanic Black, and Hispanic); fasting session index (PHDSESN); physical activity (MINMODVIG) as low versus high-activity at 150 min of moderate or vigorous activity per week; caffeine intake (DR1TCAFF), stratified as low versus high-intake at 400 mg; alcohol consumed (DR1TALCO), total calories consumed (DR1TKCAL); diabetes status (DIQ010); and moisture-intake (DR1TMOIS) stratified at males <3700 g, females <2700 g, or lactating females <3800 g. The Chang et al. study (*n* = 9601) utilized BMI (BMXBMI) as a response variable, with urine osmolality, gender, race–ethnicity, the ratio of family income to poverty level (INDFMPIR), and age as continuous variables. Data were obtained de novo from the NHANES repository at CDC.gov. These studies were selected because they provided the right balance between comparability and mutual novelty, both had already been cited multiple times in their short history in print, and both papers were written in a way that facilitated replication.

### 2.2. Model Diagnostics

A detail not reported in either study was a model goodness of fit. We extracted the model fit as R^2^ values, defined as 1 minus the ratio of residual deviance to null deviance. Our motivation for reporting R^2^ is that this is an exquisitely important parameter used to contextualize analytical models. Both papers (Chang et al. and Rosinger et al.) presented significance values (*p*-values) for individual parameters, and these values are informative as to the existence of a relationship between two variables. However, neither paper reported a goodness of fit (R^2^), so there was no way to draw an inference as to which group presented a more compelling model, or whether either model was tenable at all. A further review of models in this area of study revealed that it is the rare exception that a model goodness is published alongside the model results. Thus, there is an opportunity to provide valuable supporting information regarding our analytical approaches in the analysis of hydration datasets.

**Figure 1:**
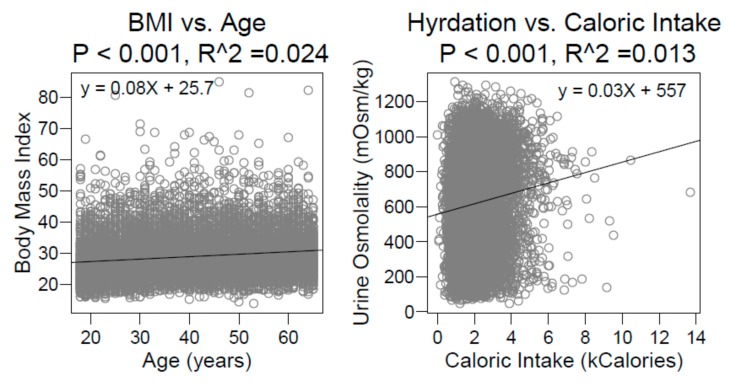
Univariate regressions from the National Health and Nutrition Examination Survey (NHANES) 2009–2010 and 2011–2012 datasets, based on Chang et al. (**Left**) and Rosinger et al. (**Right**). Both regressions have highly significant *p*-values but negligible R^2^.

In total, eight models were considered: Two from Chang et al. and six from Rosinger et al. R^2^ was low, ranging from 0.03 to 0.11. The models shown here reflect an extension of the original published analyses, starting with a reproduction of the models as originally published, using identical datasets and identical assumptions. We considered our replication successful when we were able to reproduce all linear regression coefficients described in Chang et al. to within 1% of their printed value, and we were able to reproduce all linear regression coefficients in the normal-weight dataset described in Rosinger et al., also to within 1%. Once we were able to confidently replicate these published findings by others, thus confirming their models as described, we felt comfortable extending their models. As an illustration, consider the univariate regressions shown in Figure 1. Both models yield a statistically significant relationship between predictor and response variables (both *p*-values were incalculably small, below the precision of the computer), but neither had an R^2^ above 2.5%.

We note that the relationships demonstrated in these plots depart somewhat from those reported in the prior studies due to their use of multi-variate models and weighted regressions. We have shown unweighted univariate models for the sake of clarity in visualization. Nevertheless, these figures are useful as visual aids in demonstrating the low goodness of fit in these studies. Could the weakness of these models be explained by variable configuration? We tested this in three different variables.

### 2.3. Urine Osmolality and BMI

Rosinger et al. posed hydration (via urine osmolality) as a response variable adjusted for age via the linear transformation 831 mOsm/kg − 3.4 × (age – 20 years), per published guidance [30,31], and BMI as a trichotomous predictor: Normal, overweight, and obese (BMI <25, 25–30, and ≥30). Chang et al. posed hydration as a predictor variable without adjusting for age, and they treated body composition as a dichotomous quantity: Normal versus obese stratified at BMI ≥ 30.

In order to test model sensitivity to variable configuration, we assessed the goodness of fit on the published regression models with four different settings: Hydration status with and without age adjustment and BMI as a continuous versus categorical variable. For these four models, the threshold for adequate hydration was systematically altered over a range of urine osmolality values from 200 to 1100 mOsm/kg. Thus, eight models were tested in total: Four variants from Chang et al. and four from Rosinger et al.; the BMI as a continuous versus factor variable and hydration as an adjusted versus unadjusted variable.

**Figure 2:**
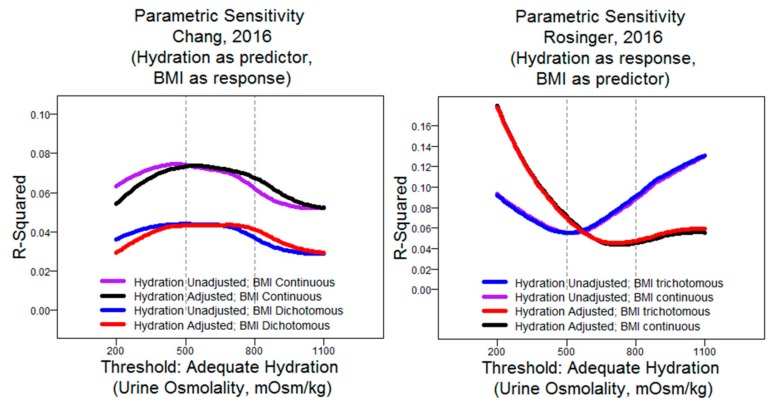
Model goodness (R^2^) versus hydration threshold (via urine osmolality) in sensitivity analysis of two database studies on hydration in relation to body composition.

We found that there were substantial differences in model fit depending on the defined threshold for adequate hydration status and that this relationship was opposite between the papers (R^2^ maximized at extreme thresholds in models derived from Rosinger et al. versus optimization in mid-range values in models derived from Chang et al.). We also noted that age adjustment seemed to have had a profound impact on the Rosinger models but not on Chang’s models—vice-versa for BMI as a continuous versus categorical measure (Figure 2). While not rigorously assessed, we suspect that this is most likely due to their respective positioning as outcome measures, as opposed to differences in datasets or inclusion of other co-variates.

A few remarks bear discussion regarding our methodology and interpretation. Firstly, Rosinger implemented separate models for each category. Here, we merged all data together and included BMI as a co-variate. While this changes the nature of the model, perturbing a single model facilitates interpretation versus three separate models, it allows for a direct contrast against Chang’s results. We specifically decided not to alter the stratification of BMI (Chang: Two-level; Rosinger: Three-level), as we felt it was valuable to retain this semblance of the published model. Additionally, we note that we intentionally tested urine osmolality ranges that were physiologically unrealistic: Stratifying at 200 mOsm/kg and 1100 mOsm/kg is unknown in the literature. While we were interested in a narrower range of strata (threshold 500–800 mOsm/kg), we felt it appropriate to test for model behavior beyond those benchmarks in order to fully describe the relationship between the model and its parameter configuration.

While it is certainly more common to consider hydration to be adequate at more moderate ranges of urine osmolality, we note that there are some respondents with values as or more extreme than this range (approximately 6% of respondents were below 200 mOsm/kg, and approximately 2% of respondents were above 1100 mOsm/kg), so while such extreme boundaries are unlikely to be useful in stratifying the general population, they are physiologically meaningful and might conceivably be of interest to those making inquiries about extreme hydration status levels. Our interest in such extreme thresholds was to explore the edge effects of the relationship between dichotomized hydration status and model goodness in order to verify that model performance is spectral and to provide perspective as to the impact of threshold selection outside of the historical range.

**Figure 3:**
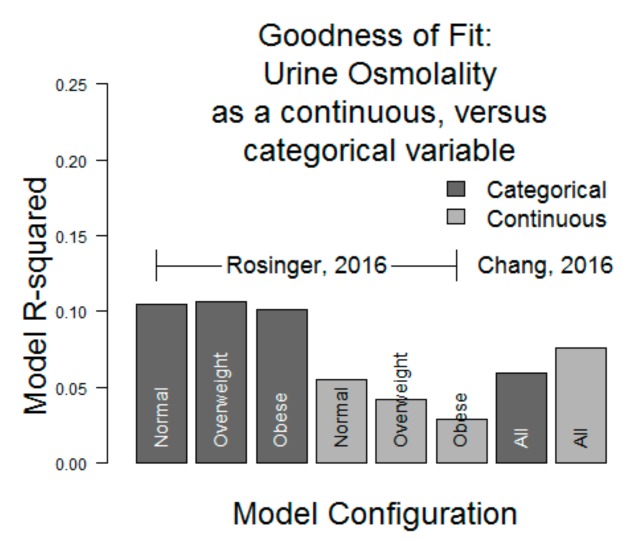
Model goodness (R^2^) comparison, incorporating hydration as a continuous variable versus categorical variable R^2^ < 0.12 in all models.

Lastly, we observed that the model fits were generally very weak: R^2^ ≤ 0.10 in all models in the interval between 500 and 800 mOsm/kg of urine osmolality. Separately, we assessed whether model fit would improve with urine osmolality as a continuous variable, and we found that the results were similar: 0.10 ≤ R^2^ ≤ 0.12 in all simulations of Rosinger’s models and 0.05 ≤ R^2^ ≤ 0.08 in Chang’s models (Figure 3).

### 2.4. Water Intake

Rosinger’s work categorizes respondents according to their water intake, with a two-level stratification, also accounting for sex (and within females: Lactation status). We tested other stratification approaches in order to test the sensitivity of the model to this parameter (Table 1).

Results were mixed: Some stratification designs yielded improved fits in the linear regressions based on Rosinger’s model, while some designs yielded a weaker fit. We note that even the best model among the 32 created yielded R^2^ < 0.14.

## 3. Discussion

### 3.1. Modeling

The papers analyzed here are not atypical in not having reported their R^2^: We are unaware of any prior database studies in hydration where model fit is reported. However, they highlight the problematic emphasis of a significance test over a heuristic and more reflective of model goodness of fit [39]. The *p*-value does not provide information about whether the data are especially adherent to the regressor. The *p*-value indicates the relationship’s existence, and R^2^ indicates the relationship’s precision.

Moreover, each point of inquiry will require its own assumptions and its own selection of response variables or outcome measures, co-variates, and data conditioning steps. It is not possible for this manuscript to serve as guidance for model design, and it is beyond the scope of this paper to critically review the designs adopted by others. Rather, we recognize the inherent variation in analytical approaches that have been published to date and that there is further variety to come. We do strongly encourage thoroughness in explaining variable selection, assumption declarations, and in reporting model goodness.

Regression models in the study of hydration face a substantial design challenge: Complex co-variate interdependencies. Total body water balance is a function of water gains (via beverages, foods and metabolic water) and losses (via sweat, urine, feces and respiratory losses). Those who expect these factors to be highly co-dependent will argue that a model containing multiple factors among this set to be poorly-posed for its inclusion of collinearities [40,41,42]. At the other extreme, those who view these factors to be acceptably independent will argue that database studies are unviable for their lack of collection of the many meaningful determinants of body water balance and that those few that are present will be inadequately supported and “over-interpreted” [43,44,45,46].

### 3.2. Asynchrony

The NHANES survey incorporates a variety of reporting windows. Dietary recall estimates consumption during the 24-h period prior to the examination center visit (midnight to midnight); physical activity reports incorporate the past seven days, past 30 days, and “in a typical week,” which may synchronize poorly with the timing of dietary information; and urine samples are collected on the day of the examination center visit, where participants are randomly assigned to appointments in the morning, afternoon, or evening, which vary somewhat versus protocols for optimal hydration assessment [38] and canonical renal function testing [47]. Investigators may or may not find this sequence (survey, delay, then measure) serviceable; others may not recognize that these are not contemporaneous measurements and may mis-interpret the models altogether.

### 3.3. Biomarkers

Tools used to indicate hydration process and status have been disputed, and no gold standard currently exists for an appropriate assessment across all scenarios [31,48,49]. While elevated urine osmolality has much perceived utility as a hydration biomarker [34,37,50,51], some question its validity with single (spot) samples [52,53]. Urine osmolality has noteworthy interindividual variation [38,53] and is an ephemeral data point which measures hydration status in an instantaneous way. However, diet, physical activity, and phenotype are more enduring. Thus, there is great need for novel biomarkers to capture this hysteresis. One emergent marker, an arginine vasopressin (AVP) surrogate, copeptin, has shown preliminary promise as a water-balance indicator with robustness to various levels of hydration status and attenuation following an increased water intake intervention [54]. Copeptin has high molecular stability compared to AVP that aids in more accurate and less complicated measurement [55], and AVP and copeptin can distinguish acute and chronic water consumption variations [37,56], rendering this circulating protein a potentially attractive option to better characterize participants’ water intake practices. While it is likely that there is no single substance or indicator with optimal responsiveness to daily hydration status [32], there is reason to continue searching for biomarkers with increasing fidelity to hydration status, as there is currently an unknown proportion of unexplained variance attributable to the biomarkers available currently.

### 3.4. Weather

NHANES currently does not collect information related to weather conditions at the time of data collection [57,58,59,60]. Seasonality corrections are common [32,61,62,63,64], although they are not universally justified [65,66], perhaps because of the complexity in adjusting for such a diffuse variable [67]. It would be feasible to document local weather conditions at or near to the time of survey response; this could be designed to capture weather in locales where the respondent habited in the preceding 24 h or to capture specific exposures, e.g., “Did you spend most of your time in a climate-controlled building or outdoors?” We observe that accounting for weather is not necessarily the sole province of future NHANES iterations: It is strictly possible to obtain zip-level geographic identifiers and date-of-survey information from restricted data (research data center) which could be cross-referenced against a historical weather database, although this requires some assumptions about a lack of travel.

### 3.5. Analytic Approach

Clearly, relationships between variables are labile to the way each variable is posed, and there are unlimited ways to create an analytical model. Should water intake be adjusted according to body composition, daily max ambient temperature, Mean temperature, or a composite measure accounting for time spent outdoors, total amount of direct sunlight, temperature and wind speed? Wearable sensors capable of recording climatic parameters are becoming increasingly prevalent [68,69,70,71], and perhaps large population investigations will soon have access to more accurate estimations of internal body temperature [72,73], sweat rate [74], and physical activity due to an increasingly robust infrastructure to support analysis of pedometers [75]. Furthermore, we recommend reporting the model goodness of fit as is conventional in modeling [76,77,78]. There are, as of yet, no published R^2^ values for hydration models in public datasets; without knowing where our benchmarks lie, it is impossible to know whether our models are keeping pace with the field in terms of explained variance. Where these actions are practicable, investigators should make efforts to incorporate them; where these actions are inappropriate, researchers should justify their approach.

## 4. Conclusions

With continued investigation into hydration practices and health outcomes, optimal model design, statistical reporting, and database extensions are warranted. By manipulating two recently-published hydration models, we were able to show (1) a substantial variation in the model fit depending on parameter configurations and (2) a consistently weak model fit, i.e., R^2^ < 0.14. We propose the inclusion of variables contributing to body water balance, which might increase the proportion of explained variance in a hydration study and mitigate artificial associations. In addition, consensus variable selection and stratification will increase comparability between studies. We make the following additional suggestions regarding targets for the advancement of the science and communication of results within the study of hydration: (1) The aggressive pursuit of promising biomarkers such as copeptin; (2) the creative utilization of assistive resources like meteorological databases; (3) the integration of advanced biostatistical techniques such as principal components analysis, survival analysis, time-series and longitudinal analysis; and (4) the detailed reporting of model goodness in any paper where modeling is employed. In particular, we recommend R^2^ and not merely a model’s *p*-value, as statistical significance is not nearly as informative as proportion of variance explained. Through the establishment of best practices and identification of new opportunities in hydration study, we anticipate that the maximum value can be obtained from database-driven inquiry, both past and future.

## Figures and Tables

**Table 1:** Goodness of fit of Rosinger et al. with various water intake stratifications used in previous publications. Cells contain R^2^ values obtained from regression models built from the published model. Top row is the replicated model from Rosinger et al.; all other rows use same dataset and same model as Rosinger et al., but with the altered stratification of a single variable (water intake). Some stratifications showed an improved goodness of fit (R^2^ greater than Rosinger et al.); some stratifications showed a degraded goodness of fit (R^2^ less than Rosinger et al.). All models showed a generally weak model fit (R^2^ < 0.15).

	Norm.	OWt	Obese	All	Strata (Water Intake (mL/day))
Rosinger, 2016 [3]	0.111	0.110	0.107	0.095	<2700 (F), <3700 (M), <3800 (Lactating F)
Armstrong, 2012 [32]	0.132	0.109	0.114	0.101	{0, 1507, 1745, 2109, 2507, 2945, 3407, ∞}
Armstrong, 2010 [33]	0.132	0.110	0.113	0.100	{0, 1382, 2008, 2048, 2453, 2614, 3261, ∞}
Johnson, 2015 [34]	0.127	0.108	0.107	0.099	{0, 1620, 3210, ∞}
Muñoz, 2015 [35]	0.126	0.106	0.111	0.099	{0, 1500, 2250, 3130, ∞}
Sontrop, 2013 [19]	0.114	0.109	0.112	0.097	{0, 2000, 4300}
Pross, 2014 [36]	0.101	0.095	0.093	0.084	{0, 1200, 2000, ∞}
Perrier, 2013 [37,38]	0.107	0.099	0.103	0.080	{0, 1200; 2000, 4000} ^1^
Roussel, 2011 [1]	0.080	0.086	0.094	0.077	{0, 500, 1000, ∞}

Norm = Normal Weight; OWt = Overweight. Strata defined or inspired by recent studies in hydration inquiry. ^1^ Middle hydration group (1200–2000 mL/d) and extremely hydrated (>4000 mL/d) censored.

## Abbreviations

AVP	Arginine vasopressin
BMI	Body-mass index
d	Day
kg	Kilogram
mL	Milliliter
mOsm	Milliosmoles
NHANES	National Health and Nutrition Examination Survey
OWt	Overweight
